# The Role of Long Non-Coding RNAs in Ovarian Cancer

**DOI:** 10.6091/.21.1.3

**Published:** 2017-01

**Authors:** Elahe Nikpayam, Behnoosh Tasharrofi, Shaghayegh Sarrafzadeh, Soudeh Ghafouri-Fard

**Affiliations:** Department of Medical Genetics, Shahid Beheshti University of Medical Sciences, Tehran, Iran

**Keywords:** Ovarian cancer, lncRNA, Biomarker

## Abstract

Ovarian cancer is the most fatal tumor of female’s reproductive system, and several genetics and environmental factors are involved in its development. Various studies have already identified some suitable biomarkers to facilitate the early detection, the prognosis evaluation, and the assessment of treatment response. However, the aim of this review is to investigate the role of long non-coding RNAs (lncRNAs) in tumorigenesis process of ovarian cancer and their potential applications as ovarian cancer biomarkers. We performed an online literature search of the MEDLINE/PubMed databases using the keywords, including ovarian cancer, lncRNA, and biomarker. We found that several lncRNAs have been shown to be deregulated in ovarian cancer and the specific mechanism of their enrollment in ovarian cancer has been defined for a few of them. In addition, expression profiling has revealed an association between lncRNAs and patients’ survival, metastasis potential, as well as treatment response. Expression profiling and methylation analysis of lncRNAs in ovarian cancer may lead to the identification of novel biomarkers that can help in the classification of patients based on prognosis and treatment response.

## INTRODUCTION

Ovarian cancer is the sixth most frequent diagnosed cancer among women worldwide, the second most common gynecologic malignancy in females, and the most fatal tumor of female reproductive system[1]. Several genetic and environmental factors have been shown to be implicated in the development of this type of cancer. For instance, estrogens participate in tumor progression by increasing cell proliferation in addition to enhancing invasion or cell mobility[^2^]. Based on histopathology, immunohistochemistry, and molecular genetic studies, malignant epithelial tumors of ovary are classified into serous carcinoma, endometrioid carcinoma, clear-cell carcinoma, and mucinous carcinoma[3]. Dysgerminoma and teratoma are also two types of germ cell tumors of ovary[4].

The International Federation of Gynecology and Obstetrics (FIGO) Committee on Gynecologic Oncology has provided a staging system for ovarian cancer[5]. The lack of specific signs and symptoms and the deficiency in screening programs have resulted in the late stage diagnosis of ovarian cancer, which in turn leads to the poor survival of these patients. Such defects have necessitated the implementation of experimental approaches and clinical studies to discover and assess biomarkers associated with early-stage disease[6].

**Fig. 1:**
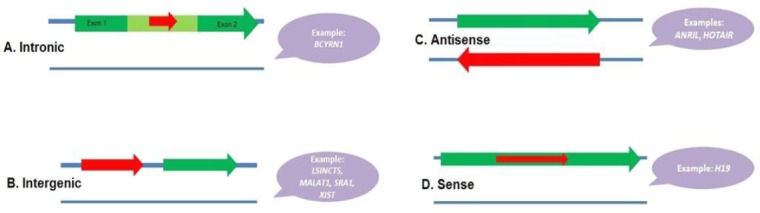
Genomic location and context of lncRNAs. Green and red colors show protein-coding genes and lncRNAs, respectively.

It has been revealed that more than 98% of the human genome encompasses non-protein-coding sequences with a substantial part of them being transcribed into non-protein-coding RNA transcripts[7]. The size of these transcripts varies from very small RNAs such as the microRNAs (miRNAs), which are 20-25 base pairs, to long non-coding RNAs (lncRNAs) that can be up to 100 kb or more[7]. LncRNAs participate in important functions of cells, including chromatin rearrangement, histone modification, and modification of alternative splicing genes, as well as the regulation of gene expression. In addition, they have been demonstrated to be involved in the processes of dosage compensation, genomic imprinting, cell differentiation, organogenesis, and tumorigenesis[7]. LncRNA can be classified based on the genomic location and context to intronic and intergenic or in close association with the mRNA genes. In latter situation, lncRNAs may be transcribed either from the same strand as the mRNA gene (sense lncRNAs) or opposite strand to make antisense lncRNAs[7] (Fig. 1). The role of lncRNAs has been assessed in different cancers and various histological tumor subtypes; besides, their differential expression has been shown in different tumor stages as well as histologic grades[7].

### Evidence acquisition

We performed an online search of the MEDLINE/PubMed, Web of Knowledge, Scopus, ProQuest, and Google Scholar databases with the key words ovarian cancer, lncRNA, and biomarker within the maximal date range until 2015.

### LncRNAs and cancer

The involvement of lncRNAs in many biological and cellular aspects such as cell proliferation, differentiation, and apoptosis suggests fundamental roles for lncRNAs in tumorigenesis processes. Further evidence for such functions has emerged from studies that validated the deregulation of lncRNAs in tumor samples compared with their normal counterparts[7]. Concerning the expression pattern and function at cellular level, lncRNAs can be classified into oncogenes, tumor-suppressor genes, and duplex lncRNAs. First category encompasses lncRNAs such as long stress-induced non-coding RNA (*LSINCT5*), metastasis-associated lung adenocarcinoma transcript 1 (*MALAT-1*), urothelial carcinoma antigen 1 (*UCA1*), Hox transcript antisense intergenic RNA (*HOTAIR*), whereas maternally expressed gene 3 (MEG3) is grouped into the second category. The H19 and steroid receptor RNA activator (*SRA)* fit in the third category as they are overexpressed in some tumors but down-regulated in other tumors[8].

### Expression pattern of lncRNAs in ovarian tumors

Ovarian cancer is among cancers in which lncRNAs expression profile has been assessed by different methods, including quantitative reverse transcription-polymerase chain reaction and high throughput techniques. LncRNAs clusters have been shown to be differentially expressed in ovarian cancer cells with distinct metastatic potentials[9]. Among 4,956 lncRNAs being analyzed in a microarray study, 583 and 578 were up-regulated and down-regulated in highly metastatic cells, compared with the parental cells, respectively, which shows a possible role for lncRNAs in epithelial ovarian cancer metastasis[9]. One study conducted in high-grade serous ovarian cancer (SOC) has identified 455 lncRNAs that were induced or repressed specifically in one of the four previously described subtypes (immunoreactive, differentiated, proliferative, and mesenchymal) relative to remaining samples[10]. Furthermore, a recent study has demonstrated that 115 lncRNAs have considerable changes in estrogen-treated ovarian cancer cells compared with untreated controls; most of them were predicted to contribute in cancer progression. In addition, it demonstrated a significant correlation of up-regulated TC0101441 and down-regulated TC0100223 and TC0101686 with estrogen receptor (ER)α+ compared to ERα ovarian cancer tissues. Additionally, the low-expression of TC0100223 and TC0101686 and the overexpression of TC0101441 have been shown to be associated with advanced FIGO stage and/or high histological grade[11]. Another recent study has shown that carboplatin-docetaxel treatment of ovarian cancer cells would result in expression change in a remarkable subset of cancer-related lncRNAs[12], indicating the possible role of lncRNAs in drug response. High levels of alpha satellite transcripts and more specifically, HSATII have been detected in epithelial cancers including ovarian cancer. Such expression patterns of satellite transcripts in cancer suggest the presence of global alterations in heterochromatin silencing, which may be considered as a cancer biomarker[13]. In the following sections, lncRNAs with remarkable participation in ovarian cancer development are presented, and the relative data are summarized in Table 1. The exact mechanism of the function for some of the presented lncRNAs in regulation of gene expression has been given in Table 2.

**Table 1:** Long non-coding RNAs expression in ovarian cancer

LncRNA	Chromosomal location	Expression pattern in ovarian cancer	Involvement in other cancers	Function/characteristics	Method of identification in ovarian cancer	Ref.
*ANRIL*	9p21.3	UP	Prostate, melanoma, breast, pancreatic carcinoma, nasopharyngeal carcinoma, basal cell carcinoma, glioma and leukemia	Regulates its neighbor tumor suppressors *CDKN2A/B* by epigenetic mechanisms & regulate cell proliferation	qRT- PCR	[14]
*AB073614*	N/A	UP	-	Involves in cell proliferation and invasion	qRT-PCR	[15]
*BCYRN1 (BC200)*	2p21	UP	Cervical, lung, esophageal, breast, parotid, tongue	Is a molecular indicator of invasive malignancy	Northern hybridization	[16]
*CCAT1*	8q24.21	UP	Colorectal, gastric, breast, prostate, gallbladder, hepatocellular carcinoma	Involves in cell proliferation and migration	Microarray analysis, qRT-PCR	[9]
*DNM3OS*	1q24.3	DOWN	-	Unkown	qRT-PCR	[17]
*FAL1*	1q21.2	UP	Lung, breast, liver	Promotes cancer cell growth via repression of *p21*, regulates the transcription of *CDKN1A* via stabilization of *BMI1*	Functional genomic approach	[18]
*GAS5*	1q25.1	DOWN	Kidney, breast lymphoma, prostate	Induces growth arrest and apoptosis	Functional study	[19]
*H19*	11p15.5	UP	Bladder, cervical, colon, esophageal, gastric, glioblastoma, hepatocellular, lung, breast, prostate, melanoma, meningioma, adrenocortical carcinoma	Is essential for tumor growth	Northern hybridization, functional study, microarray analysis, qRT-PCR	[20]
*HOST2*	10q23.1	UP	-	Promotes tumor cell migration, invasion and proliferation	qRT-PCR	[21]
*HOTAIR*	12q13.13	UP	Colorectal, cervical, endometrial, gastric, squamous cell, gastro-intestinal, hepatocellular, liver, lung, pancreas, small cell lung cancer, breast	Involves in cancer invasiveness	Massively parallel sequencing	[22]
*HOXA11-AS*	7p15.2	DOWN	Cervical cancer	Acts as a tumor suppressor gene	semi-quantitative PCR	[23]
*LOC554202*	N/A	UP	Breast	Regulates proliferation and migration	qRT-PCR	[24]
*LSINCT5*	5p15.33	UP	Breast	Unknown	Microarray	[25]
*MALAT1*	11q 13.1	UP	Bladder, cervical, endometrial, colorectal, hepatocellular, kidney, liver, lung, neuroblastoma, non-small cell lung cancer, osteosarcoma, pancreas, prostate, uterus, breast	Plays a critical role in pre-mRNA alternative splicing	In-situ hybridization	[26]
*MEG3*	14q 32.2	DOWN	Leukemia, bladder, colon, gastric, glioma, hepatocellular, kidney, lung, meningioma, neuroblastoma, prostate, breast	Unknown	Northern hybridization, RT-PCR	[27]
*NEAT-1*	11q13.1	DOWN	Oral squamous cell carcinoma	Acts as a transcriptional regulator for numerous genes, including some genes involved in cancer progression	qRT-PCR	[25]
*NRCP*	3q23-q25	UP	N/A	Involved in apoptosis, cell proliferation and glycolysis	qRT-PCR	[28]
*OVAL*	1q25	UP	-	Unknown	qRT-PCR	[10]
*PVT1*	8q24	UP	Burkitt and Hodgkin’s lymphoma, breast, pancreas, prostate, renal	Acts as a *MYC* activator, involved in cell proliferation and apoptosis	qRT-PCR, Functional study	[12,29]
*SRA1*	5q31	UP	Breast, uterus	Acts as transcriptional co-activator of steroid hormone receptors	qRT-PCR	[30]
*UCA1*	19p13.12	UP	Bladder, oral squamous cell carcinoma, breast, gastric cancer, tongue squamous cell carcinomas	Suppresses the *p27* protein level in interaction with hnRNP I	qRT-PCR, functional study	[9]
*XIST*	Xq13.2	DOWN	Bladder, testicular, breast, female cancers,	Acts in X chromosome inactivation	qRT-PCR	[31]
*ZNF300P1*	5q33.1	DOWN	-	Involves in cell proliferation and cellular polarity	Methylation analysis, qRT-PCR	[32]

Ref., reference, UP, up-regulation; DOWN, down-regulation

**Table 2:** The results of functional studies demonstrating the role of lncRNAs in gene expression regulation

LncRNA	Transcriptional regulation		Function	
	
	*Cis*	*Trans*	Translational regulation	Splicing regulation
*ANRIL*	✓			
*BCYRN1*			✓	
*GAS5*		✓		
*H19*	✓			
*HOTAIR*		✓		
*HOXA11-AS*	✓			
*MALAT1*				✓
*MEG3*	✓			
*XIST*	✓			

### AB073614

AB073614 is a functional oncogene in the process of ovarian cancer development. It has been shown to be up-regulated in 85.3% of ovarian cancerous tissues compared with normal counterparts[15]. Besides, patients with high expression of AB073614 had a lower 5-year overall survival in comparison with low expression group. Down-regulation of this lncRNA using small interfering RNA (siRNA) strategy in ovarian cancer cell lines considerably reduced cell proliferation and invasion, leading to cell arrest in G1 phase of cell cycle and a remarkable increase in apoptosis. The function of this lncRNA may be exerted through the targeting of extracellular signal-regulated kinase (ERK)1/2 and Protein Kinase B Alpha (PKB, AKT)-mediated signaling pathway[15].

### Antisense non-coding RNA in the INK4 locus (ANRIL)

*ANRIL* have been recently shown to be located in the region of 9p21, which is a hotspot for disease-associated polymorphisms[33]. It regulates the expression of its neighboring tumor suppressors *CDKN2A/B* through epigenetic modulation. Besides, elevated *ANRIL* expression restrains the expression of *INK4a*, *INK4b*, and *ARF* at the late-stage of DNA damage response. A recent study has demonstrated its up-regulation in malignant breast tissues compared with adjacent non-cancerous tissues with a higher expression in estrogen receptor, progesterone receptor, and HER2 triple-negative samples[33]. Another recent study has shown the overexpression of ANRIL in SOC tissues compared with normal controls and its correlation with advanced FIGO stage, high histological grade, lymph node metastasis, and poor prognosis[14]. It has also been suggested that *ANRIL* can be used as an independent prognostic factor for the prediction of overall survival of SOC patients, and it is significantly overexpressed in highly metastatic ovarian cancer sublines[14]. *In vitro* study has confirmed its important role in SOC invasion/metastasis and has shown *MET* and *MMP3* as key downstream genes of *ANRIL* in this process[14].

### Brain cytoplasmic RNA 1, BC200 (BCYRN1)

BCYRN1 is an lncRNA with selective expression in the primate nervous system, specifically in somatodendritic domains of a subset of neurons. Its RNA is not normally expressed in non-neuronal somatic cells. Expression analysis of BCYRN1 in several tumor types has revealed that this lncRNA is expressed in tumor tissues including ovarian cancer samples, but not in the corresponding normal tissues[16].

### Colon cancer associated transcript 1 (CCAT1)

As a newly discovered lncRNA, *CCAT1* is located near the well-known cancer-related gene, *c-Myc*, on the 8q24 region. The expression of this lncRNA can be induced directly by *c-Myc* binding to *CCAT1* promoter region. The elevated expression level of *CCAT1* in gastric cancer cell lines enhances cancer cell proliferation and migration[34]. CCAT1 is among lncRNAs with differential expression in ovarian cancer cells with varying metastatic potentials. As siRNA-mediated *CCAT1* silencing has resulted in decreased invasion ability of ovarian cancer cells, it can be deduced that *CCAT1* potentially enhances the invasion ability of these cells[9].

### Focally amplified lncRNA on chromosome 1 (FAL1)

FAL1 is a lncRNA identified by a functional genomic approach. FAL1 RNA overexpression has been shown to be a common event in cancer cells[18]. In addition, its expression has been detected in more than 93% of the ovarian cancer samples. A nuclear-enriched staining pattern, with a weak signal in cytoplasm has been detected in both FAL1-positive samples and cancer cell lines[18]. In addition, FAL1 RNA expression and genomic copy number have been shown to be higher in late-stage than early-stage tumors[18]. Besides, both higher expression of FAL1 RNA and genomic gain of *FAL1* gene were notably associated with reduced survival in patients. The oncogenic activity of *FAL1* has been attributed to its inhibitory effect on *p21*[18]. Furthermore, *FAL1* associates with the epigenetic repressor *BMI1* and controls its stability in order to change the transcription of a number of genes including *CDKN1A*[18].

### Growth arrest-specific transcript 5 (GAS5)

GAS5 expression has been shown to be lower in epithelial ovarian cancer tissues compared with normal ovarian epithelial tissues; however, no difference has been found between normal ovarian epithelium and benign epithelial lesions[19]. Its down-regulation has been supposed to be associated with lymph node metastasis and tumor node metastasis stage[19]. Furthermore, exogenous GAS5 suppressed proliferation, enhanced apoptosis, and decreased migration, and the invasion of ovarian cancer cells. It is also able to disrupt mitochondrial membrane potential and enhances BAX, BAK, cleaved-caspase-3 and -9 expressions. Consequently, it has been suggested as a novel therapeutic target in patients with epithelial ovarian cancer[19].

### H19

*H19* is an oncofetal gene highly expressed in fetal tissues, but repressed in most tissues in postnatal period. In addition to encoding for a lncRNA, it is a precursor for miR-675, which modulates the expression of genes crucial for growth, development, and carcinogenesis including Retinoblastoma (RB)[35,36]. Overexpression of H19 has been linked with enhanced proliferation, tumorigenesis, cell cycle progression, and cell migration[37,38]. Its role in enhancing tumor cell migration and invasion has been shown to be mediated by inhibiting let-7, a tumor suppressor miRNA that down-regulates the expression of oncogenes modulating cell growth and motility[39]. *In vivo* studies have also shown the co-expressions of oncogenes and *H19* in both primary human ovarian and endometrial cancers, confirming the H19/let-7-dependent regulation[39]. Notably, the anti-diabetic drug, metformin, has been shown to suppress the tumor cell migration and invasion, partly by epigenetic down-regulation of *H19*[39]. The frequent loss of *H19* imprinting has been detected in ovarian cancer tissues, especially in malignant serous cystadenocarcinomas[40]. H19 is expressed in the majority of serous epithelial tumors, which may suggest the possible application of this lncRNA as an adjuvant tumor marker in diagnosis, staging, and follow-up of patients with such disorders[20]. It is also up-regulated in most ovarian cancer tissues compared with adjacent non-tumor samples with a significantly positive correlation between its expression and tumor stages and tumor size[41]. In addition, it is among lncRNAs with differential expression in ovarian cancer cells with varying metastatic potentials[9]. *H19* knockdown has been shown to inhibit the growth and clonogenicity of epithelial ovarian cancer cells in a synergic manner with histone H1.3 overexpression[42]. One study has revealed that the silencing of H19 would lead to the induction of cell apoptosis and cell cycle arrest at the G2/M phase[41]. Besides, *H19* RNA has been detected in the majority of patients with ovarian cancer ascites fluid. The intratumoral injection of diphtheria toxin A chain-*H19* into ectopically developed tumors has led to a significant inhibition of tumor growth[43].

### Human ovarian cancer-specific transcript 2 (HOST2)

The lncRNA HOST2 contains multiple copies of retroviral-related sequences and has been identified through serial analysis of gene expression (SAGE) of ovarian cancer samples[44]. Although it is infrequently expressed in normal tissues or non-ovarian cancers, its expression has been commonly demonstrated in ovarian cancer-derived cell lines and primary tumors. Furthermore, it is up-regulated in all four major subtypes of ovarian cancer compared to cultivated ovarian surface epithelial cells[44]. In addition, *HOST2* has been reported to enhance tumor cell migration, invasion, and proliferation in epithelial ovarian cancer, possibly by the inhibition of miRNA let-7b tumor suppressor functions[21].

### HOX transcript antisense RNA (HOTAIR)

HOTAIR is a long intervening non-coding RNA (lincRNA) transcribed from the *HOXC* locus, which participates in epigenetic regulatory processes, cooperates with polycomb repressive complex 2 and is required for histone H3 lysine-27 trimethylation of the *HOXD* locus. Its expression has been significantly associated with the invasion and metastasis of cancer cells[45]. Its expression has also been shown to be higher in epithelial ovarian cancer tissues than in the benign ovarian tissues, in late-stage malignant ovarian tumors compared with the early-stage tumors. In addition, it is overexpressed in the SKOV-3 CDDP/R cisplatin-resistant ovarian carcinoma cell line than in the SKOV-3 cisplatin-sensitive cell line. Its knockdown inhibited cell proliferation, decreased the invasion ability of the cells and restored the cisplatin sensitivity of the cisplatin-resistant cells of corresponding cells[46]. Furthermore, it has been shown that it is involved in mesenchymal stem cell fate[47]. Its expression has been demonstrated to be higher in ovarian cancer stem cell (CSCs) than in non-CSCs[47]. Considering the role of CSCs in tumorigenesis and drug resistance[48], it can be a target for anti-cancer therapies. The down-regulation of HOTAIR has resulted in a marked decrease in CSC migration and invasion and significantly diminished the tumor growth and lung metastasis in xenograft mice. Consequently, this strategy has been suggested as a promising novel modality for future clinical trials[49]. HOTAIR is overexpressed in SOC tissues compared with normal controls. Its level of expression can predict overall survival in these patients[22]. The CSC level of expression has been associated with an advanced FIGO stage and a high histological grade. Its knockdown has induced cell cycle arrest and apoptosis in ovarian cancer cell lines[22]. One study has revealed that its expression and surrogate DNA methylation signature have been significantly associated with poor survival in carboplatin-treated ovarian cancer patients and predict carboplatin resistance in ovarian cancer patients[47]. Consequently, it has been suggested as a marker for individualized treatment and a new target to defeat carboplatin resistance[47].

### Homeobox A 11 antisense (HOXA11-AS)

An exonic variant within *HOXA11-AS*, rs17427875 (A>T), has been shown to be slightly associated with reduced SOC risk[23]. In addition, the expression of minor allele T in epithelial ovarian cancer cells has been related to decreased proliferation, migration, and invasion compared to common allele A. Additionally, the stable expression of *HOXA11-AS* minor allele T reduced the primary tumor growth in mouse xenograft models, to a greater extent, than common allele A. Besides, *HOXA11-AS* expression levels were considerably lower in ovarian cancer tissues compared with normal ovarian tissues, implying a tumor suppressor role for this lncRNA in ovarian cancer, which may be increased by the T allele[23].

### Long stress-induced non-coding transcript 5 (LINCT5)

LINCT5 has been shown to be overexpressed in breast and ovarian cancer cell lines and tumor tissues, compared with their normal counterpart[25]. Its expression has been demonstrated to be 5-fold to 26-fold greater in primary ovarian tumors in comparison with normal tissue. Additionally, its silencing in cancer-derived cell lines led to down-regulation of chemokine (C-X-C motif) receptor 4 (CXCR4) and reduced cell proliferation. Its role in cellular processes has been validated by high-throughput expression analysis, which shows that its knock down significantly down-regulates the expression of tens of genes including the lncRNA nuclear paraspeckle assembly transcript 1 (*NEAT-1*) and the protein-coding gene paraspeckle component 1[25].

### Metastasis-associated lung adenocarcinoma transcript 1 (MALAT1)

As one of the first identified cancer-associated lncRNAs, MALAT1 contributes to the pathogenesis of many types of tumors, including hepatocellular carcinoma, cervical cancer, breast cancer, ovarian cancer, and colorectal cancer[26]. MALAT1 knockdown has suppressed the proliferation and the invasion of human osteosarcoma cell and inhibited its metastasis potential. *MALAT1* function has been shown to be mediated through PI3K/AKT signaling pathway[26]. In addition, the expression of this lncRNA has been considerably increased in primary bladder tumors with subsequent metastasis, but not in non-metastasized tumors. Its silencing has resulted in a decrease in the epithelial-mesenchymal transition (EMT)-associated Zinc Finger E-Box Binding (ZEB) 1 and 2, and Slug levels, as well as an increase in the E-cadherin levels in bladder cancer cells. The role of *MALAT1* in EMT enhancement in these cells has been shown to be mediated by activating the Wnt signaling[50]. Although its mechanism of action in ovarian cancer has not been elucidated yet, it has been shown to be among lncRNAs with differential expression in ovarian cancer cells with varying metastatic potentials[9], which is in accordance with its metastatic role in other cancers.

### Maternally expressed gene 3 (MEG3)

*MEG3* is a lncRNA that can activate p53 and prevents tumorigenesis and development of many types of cancers. However, its expression has been shown to be decreased or abolished in most epithelial ovarian cancer tissues and cell lines due to intensive promoter hypermethylation[27]. In addition, ectopic expression of *MEG3* has inhibited the proliferation and the growth of ovarian cancer cells and enhanced their apoptosis. Consequently, it has been deduced that *MEG3* promoter hypermethylation may contribute to the development of epithelial ovarian cancer[27].

### Nuclear paraspeckle assembly transcript 1(NEAT1)

NEAT1 is necessary for the structure of nuclear paraspeckles and is up-regulated in ovarian cancer[51]. Considerably, it is among those lncRNAs that are being repressed in the proliferative subtype of high-grade SOC[10]. *NEAT1* levels secreted by adipose-derived stem cells (ADSC) from morbidly obese patients have been significantly higher than those from normal ADSC. It has been shown that tumor migration of ovarian cancer cells is increased when co-cultured with ADSC of obese patients[52]. Additionally, its silencing in these ADSCs has resulted in decreased tumor cell migration of ovarian cancer cells. Consequently, it has been concluded that the secretion of *NEAT1* is increased in the setting of obesity to strengthen the tumor niche[52].

### Non-coding RNA ceruloplasmin (NRCP)

NRCP participates in cancer cell glycolysis and promotes tumor growth and proliferation. It has been shown to be significantly up-regulated in ovarian tumors[28]. Unlike control cancer cells, the knockdown of *NRCP* increased apoptosis, decreased cell proliferation, decreased glycolysis, and reduced migration. In addition, siRNA-mediated silencing of *NRCP* has reduced tumor growth in an orthotopic mouse model of ovarian cancer[28].

### Ovarian adenocarcinoma amplified lncRNA (OVAL)

OVAL is an intergenic lncRNA located on chromosome 1. It shows narrow focal genomic amplification in a subset of tumors including high grade SOC and uterine corpus endometroid carcinoma; however, no obvious focal signal has been detected in other cancers[10]. Interestingly, endometrial tumors of the serous subtype were more likely to carry *OVAL* focal amplification compared to non-serous tumors. Consequently, *OVAL* amplification is detected specifically in serous tumors regardless of tumor site[10].

### Plasmacytoma variant translocation 1 (PVT1)

*PVT1* resides in the 8q24 genomic region in the neighborhood of *c-Myc*. Genomic rearrangements and amplifications have been frequently demonstrated in hematological malignancies and solid tumors, respectively[29]. *PVT1* has a *c-Myc*-independent role in ovarian and breast pathogenesis when overexpressed due to genomic abnormalities. The inhibition of PVT1 expression in overexpressing cell lines has remarkably resulted in apoptotic response. This type of response has not been observed in cell lines lacking the PVT1 amplification/overexpression[29]. One study has shown the frequent copy number alterations in 8q region in ovarian cancer samples[53]. Interestingly, there was overexpression of *PVT1*, but not *MYC*, in these samples compared to tumors lacking this copy number alteration[53], which implies a significant contribution of this lncRNA in ovarian cancer development. In addition, its expression is induced by the mixture of carboplatin and docetaxel. Additionally, it participates in anti-cancer activity of this combination chemo-therapy, possibly by increasing the expression of *p53* and tissue inhibitor of matrix metalloproteinases-1[12].

### Steroid receptor RNA activator 1 (SRA1)

SRA1 has been primarily identified as a non-coding RNA gene, but it is alternatively transcribed to a protein-coding RNA as well[54]. It is a nuclear coactivator of steroid hormone receptors as well as non-steroid nuclear receptors and other transcription factors[54,55]. Its overexpression has been demonstrated in a significant percentage of ovarian carcinomas and in a positive association with the size of the tumor, the grade and the stage of the disease, and the debulking success. Lower *SRA1* expression increases the patients’ overall survival and the progression free survival[30]. Consequently, it can be considered as an independent prognostic biomarker in ovarian cancer[30]. In addition, it is among those up-regulated genes in post chemotherapy ovarian cancer samples, which is related to tumorigenesis and conntributes to chemo-resistance phenotype[56].

### Urothelial carcinoma antigen 1 (UCA1)

UCA1 is a regulator of cell growth in bladder carcinoma[57] and has been shown to be up-regulated in breast cancer[7]. It is among lncRNAs with differential expression in ovarian cancer cells with varying metastatic potentials[9]. In addition, it has been claimed to be repressed in the proliferative subtype of high-grade SOC[10]. An investigation has shown the elevated expression of UCA1 in ovarian cancer tissues and revealed that the expression of UCA1 RNA in SKOV-3 cells could increase the cell migration, invasion, and cisplatin resistance of these cells. Such function of UCA1 has been shown to be mediated via SRPK1 and apoptosis pathway proteins[58].

### X-Inactive Specific Transcript (XIST)

*XIST* encodes a spliced lncRNA with a unique characteristic of being expressed solely from the inactive X chromosome[31]. It has been shown that XIST is involved in the X chromosome inactivation process. In a study aimed at comparing the total RNA expression profiles between primary and recurrent ovarian tumors from the same patient, XIST was the most differentially expressed gene that was down-regulated in the recurrent tumor[31]. In addition, *in vitro* studies showed that its expression level correlates considerably with Taxol sensitivity. Furthermore, a strong association has been found between XIST RNA levels and disease-free periods of ovarian cancer patients, who received Taxol in their therapeutic regiments. The loss of inactive X chromosome has been suggested as a mechanism for the loss of *XIST* transcripts in the ovarian cancer cell lines. It has been proposed that the down-regulation of *XIST* acts as an underlying mechanism for the up-regulation of X-linked inhibitor of apoptosis and the prevention of drug-induced apoptosis, which leads to resistance phenotype in cancer cells[31]. Additionally, since the loss of inactive X chromosome is a frequent event in cancer cells, the absence of XIST expression may be a marker for genetic instability correlated with drug resistance[31]. Another study has revealed that the dysregulation of X chromosome inactivation is common in high-grade SOC and is associated with poor prognosis in these patients[59].

### ZNF300P1 (LOC134466)

*ZNF300P1* has been characterized as a pseudogene of the human zinc finger protein ZNF300 (sharing 89% identity)[32]. Its promoter has been shown to be frequently hypermethylated and silenced in ovarian cancer tissues[32]. ZNF300P1 has also been demonstrated to be involved in the regulation of important cell cycle and cell motility networks in human ovarian surface epithelial cells and may participate in promoting metastasis in ovarian cancer cells[32]. Furthermore, its down-regulation leads to decreased cell proliferation and colony formation in addition to abnormal and less persistent migration because of the loss of cellular polarity. It is also involved in the attachment of ovarian cancer cells to peritoneal membranes, suggesting a probable function of ZNF300P1 expression in metastasis of ovarian cancer cells to sites within the peritoneal cavity[32].

## DISCUSSION

The data presented above shows that numerous lncRNAs exhibit deregulation in ovarian cancer. Such expression pattern has provided a potential for them to be used as cancer biomarkers at mRNA level. Another approach would be the evaluation of epigenetic changes in the promoter region of lncRNAs, which facilitates the discrimination of malignant tissues from normal counterparts as documented for a potential long-intergenic non-coding RNA gene (*LOC134466*) in SOC[60]. LncRNA expression profiling should be assessed in each cancer type as it has revealed that the most altered lncRNAs are different in distinct cancers[61]. Additionally, they may facilitate differentiation between ovarian cancer histologic subtypes due to difference in their expression pattern among different subtypes[10]. Collectively, the data presented above indicate that lncRNAs have the potential to be used as sensitive and specific biomarkers for the identification of disease prognosis and designing specific approaches to prevent tumor growth or metastasis based on expression profile. LncRNA-based treatment modalities are predicted to essentially improve the treatment and the prognosis of ovarian cancer[62]. The global expression analysis of lncRNAs and mRNAs in ovarian cancer using microarray and the assessment of their interrelationship via co-expression analysis would be an effective strategy for identification of novel biomarkers as well as understanding the tumorigenesis process.

Although several lncRNAs have been shown to be deregulated in ovarian cancer, the data regarding the mechanism of lncRNAs function in cell proliferation, apoptosis, and metastasis are scarce. However, the level of lncRNAs expression is associated with disease prognosis, patients’ survival, and therapeutic response[14,15]. In addition, their expression has been found to be significantly regulated by estrogen. Considering the role of estrogen in ovarian cancer development, lncRNAs present a new field for researches to identify new targets for cancer treatment. Furthermore, as they are implicated in drug resistance in ovarian cancer patients, their expression profiling is a novel approach to determine new molecular targets for cancer pharmacology[31]. Other possible treatment modality would be targeting the expression of a putative toxin gene under the control of an lncRNA regulatory sequence in ovarian tumor cells[44,63]. The expression profiling of tumor samples permits the prior identification of non-responders and avoidance of treatment failure.

### Future perspectives

Based on the recent rapid flow of information regarding the role of lncRNAs in the development of ovarian cancer, it is anticipated that the expression profiling of these non-coding RNAs helps in the identification of molecular biomarkers for the early detection of this cancer. The advent of next-generation sequencing tools for lncRNAs quantification and profiling has paved the way for such biomarker discovery. The diverse and wide range of lncRNAs function in tumor biology implies that future targeted therapies against lncRNAs will improve the survival rate of patients suffering from this type of cancer.
